# Microglial Phenotypes and Their Relationship to the Cannabinoid System: Therapeutic Implications for Parkinson’s Disease

**DOI:** 10.3390/molecules25030453

**Published:** 2020-01-21

**Authors:** Rachel Kelly, Valerie Joers, Malú G. Tansey, Declan P. McKernan, Eilís Dowd

**Affiliations:** 1 Pharmacology & Therapeutics, National University of Ireland, H91 W5P7 Galway, Ireland; r.kelly35@nuigalway.ie (R.K.); declan.mckernan@nuigalway.ie (D.P.M.); 2 Department of Neuroscience, University of Florida College of Medicine, Gainesville, FL 32611, USA; vjoers@ufl.edu (V.J.); mgtansey@ufl.edu (M.G.T.); 3 Center for Translation Research in Neurodegenerative Disease, University of Florida College of Medicine, Gainesville, FL 32611, USA

**Keywords:** neuroinflammation, microglia, phenotypes, cannabinoids, neurodegeneration, Parkinson’s

## Abstract

Parkinson’s disease is a neurodegenerative disorder, the motor symptoms of which are associated classically with Lewy body formation and nigrostriatal degeneration. Neuroinflammation has been implicated in the progression of this disease, by which microglia become chronically activated in response to α-synuclein pathology and dying neurons, thereby acquiring dishomeostatic phenotypes that are cytotoxic and can cause further neuronal death. Microglia have a functional endocannabinoid signaling system, expressing the cannabinoid receptors in addition to being capable of synthesizing and degrading endocannabinoids. Alterations in the cannabinoid system—particularly an upregulation in the immunomodulatory CB_2_ receptor—have been demonstrated to be related to the microglial activation state and hence the microglial phenotype. This paper will review studies that examine the relationship between the cannabinoid system and microglial activation, and how this association could be manipulated for therapeutic benefit in Parkinson’s disease.

## 1. Introduction

Microglia are the resident mononuclear phagocytes of the central nervous system (CNS) and are found ubiquitously throughout the brain and spinal cord. Depending on the region, they comprise 5–12% of the total glial cells in the rodent brain [1], and 0.5–16.6% of glial cells in the human brain [2]. Microglia play two primary roles in the brain and spinal cord: in immune defense, and in maintenance of CNS homeostasis. As part of the innate immune system, microglia act as sentinels, constantly scouring the environment of the CNS for the first signs of danger, such as pathogens or tissue damage [3,4,5,6]. Detection of such signals initiates a cascade of responses that aim to resolve the injury. However, if chronic microglial activation persists, it can have a detrimental effect on the preservation of homeostasis in the CNS and can thus contribute to disease, as is the case with age-related neurodegenerative disorders such as Parkinson’s disease (PD). Cannabinoid receptors have been shown to be present on microglia and when activated can modulate the inflammatory profile of these cells [7,8]. Therefore, in recent years there has been a piqued interest in the potential of cannabinoids as a therapeutic tool to limit the detrimental effects of neuroinflammation and the associated neuronal death in neurodegenerative diseases such as PD.

## 2. Microglia

### 2.1. The Origin of Microglia: A Historical Perspective

Microglial cells were first observed in the central nervous system in the late 19th century, when psychiatrist Frank Nissl noted the presence of rod cells or ‘stäbchenzellen’ and described them as reactive neuroglia with phagocytic and migratory potential [9]. Ramón y Cajal later developed an improved cell staining method using gold chloride sublimate, which he was able to use to excellently stain astrocytes but which also poorly stained small adendritic cells that seemed devoid of processes. He termed these cells the ‘third element’ of the central nervous system [10], recognizing them to be different from neurons and astrocytes, the first and second elements respectively. However, it was Pío del Río-Hortega who built on Cajal’s work and introduced novel staining methods, which allowed him to separate this ‘third element’ into two distinctive cell types: microglia and ‘interfascicular cells’, the cells that we now know as oligodendrocytes [11]. Río-Hortega is considered the father of microglia and was the first one to use the term to describe these cells [12]. Many of his initial observations on microglia are still relevant to this day. In four landmark papers in 1919 [13,14,15,16], he described the morphology of microglia in normal tissue and reported their high phagocytic and migratory activity in conditions where tissue damage had occurred. He also presented evidence that they transformed into the cells that had previously been seen in pathological tissue but whose origin had been heavily debated. He hypothesized that microglia were of mesodermal origin, contrary to the commonly held belief at the time that all glial cells were of neuroectodermal origin [17]. The origin of microglial cells remained an ongoing discussion over the ensuing decades, persisting until recent years. Two principal hypotheses existed, supporting contrastingly the neuroectodermal and mesodermal origins of these cells.

The neuroectodermal origin hypothesis was based on the presumption that microglia shared a common progenitor with oligodendrocytes and astrocytes, the other glial cells that exist in the brain, a theory that subsequently gathered considerable evidence and support [18,19,20]. These cells were all proposed to arise from free subependymal cells (glioblasts), which originated from the neuroectoderm. Indeed, one ultrastructural study described the existence of cells with intermediate features between a glioblast cell and a microglial cell [21]. Autoradiographic analyses also indicated that microglia originated from glioblasts. Brain tissue from mice at various stages of postnatal development, which had been injected with ^3^H-thymidine, showed that 91% of glial cells in the hippocampus were produced after birth. The researchers observed a continuous morphological transition from proliferating cells (which retained similar structural characteristics to glioblasts at late postnatal days) to resting microglia [22]. Comparable results were also obtained using a similar technique in rats [23].

The mesodermal origin hypothesis was based on the clear phenotypic similarities of microglia to peripheral monocytes/macrophages, and a number of studies using various techniques did conclude that microglia were derived from bone marrow stem cells [24,25,26]. Immunohistochemical studies determined that microglia expressed specific macrophage markers, such as MAC-1, F4/80 and the Fc receptor in mouse microglia [27], and FcγRI and CD11b in human microglial cells [28]. However, a large number of members of the scientific community believed that microglia were derived from circulating monocytes. This belief was supported by studies that showed engraftment of the CNS by CCR^2+^ monocytes after myeloablation using irradiation [29,30]. However, irradiation severely impacts the viability of microglia, so this may not accurately demonstrate microglial turnover in homeostatic conditions [31]. In the mature adult brain, although there is varying recruitment of bone marrow progenitors in disease states [32,33,34], in normal, healthy conditions the microglial population can be replenished by self-renewal in a stochastic process, rather than from de novo hematopoietic progenitors [17,35,36,37].

The concept that microglia develop from the embryonic yolk sac was first suggested in the 1990s by Cuadros and co-workers [38], whose study in avian embryos showed that primitive myeloid cells entered the brain rudiment during development, and that these cells were of yolk sac origin. Alliot and colleagues [39] provided further evidence to support this hypothesis, as they showed microglial progenitors to be present in the yolk sac of the developing mouse embryo, and later in the brain rudiment from embryonic day 8. In 2010 yet more definitive evidence was provided by Ginhoux et al. [40], who used in vivo fate mapping in mice to show that adult microglia are derived from primitive macrophages, which arise from the embryonic yolk sac during development, and enter the brain rudiment via the circulatory system. Another group that likewise used fate-mapping techniques in mice also confirmed the results suggesting that the origin of these cells is from the yolk sac [41]. It is now generally accepted by the scientific community that microglial cells do indeed arise from the embryonic yolk sac. The first wave of production of early primitive macrophages and erythrocytes occurs at embryonic day 8.5 (E8.5) in the murine yolk sac and has been described as part of a transient process termed ‘primitive hematopoiesis’ differentiating it from a second wave of production of definitive hematopoietic stem cells (HSCs), which occurs around E10.5, known as ‘definitive hematopoiesis’. These HSCs are generated in an area surrounding the dorsal aorta of the embryo, termed the aorta-gonad-mesonephros (AGM) region, and then migrate to the fetal liver and bone marrow to differentiate into all hematopoietic lineages [42,43,44,45]. This is in contrast to microglia that originate from the first wave of primitive myeloid progenitors and colonize the brain.

Blood circulation is critical for the seeding of the central nervous system by the microglial progenitors, which is demonstrated by the absence of microglia in mice devoid of a heartbeat and therefore functional circulation due to a sodium calcium exchanger 1 deficiency [40]. In mice, formation and remodeling of blood vessels occur between embryonic days 8 and 10 [46], at which time the vasculature changes from a simple loop into a complex three-dimensional organ. Initial gradual infiltration of the microglial progenitors into the neuroepithelium of mice begins from E8–E14, followed by a rapid infiltration with a massive increase in microglia from E14–E16. This is followed by another gradual infiltration stage, lasting until E17.5, during which the progenitors are also dispersing throughout the brain [47].

During neonatal development, microglial progenitors do not resemble microglia found in the healthy adult brain. They proliferate and gain the highly ramified processes typical of mature resting microglial cells through a series of developmental steps. Interestingly, considerable sex differences have been found with regards to microglia before and after development including their number and morphology; the long-term consequences of these differences on the immunological function has not been fully elucidated [48]. Once adult ramified microglia are spread throughout the CNS, a number of factors contribute to their self-renewal, ensuring maintenance of microglial cell numbers and thus maintenance of CNS homeostasis [49].

### 2.2. Microglial Phenotypes and Activation

Microglia execute a variety of crucial functions in the CNS. They play critical roles in the maintenance of synaptic plasticity, especially postnatally and in early adulthood. Paolicelli and colleagues showed that they play an important role in remodeling neural circuits postnatally in mice, as they actively engulf synaptic material and undertake synaptic pruning [50]. They also regulate adult neurogenesis [51,52], promote learning-dependent synapse formation [53], phagocytose dead or dying cells and debris [54,55], and maintain myelin homeostasis [56,57,58]. In addition to their housekeeping functions, microglia are imperative to the immune response, mediating host defense against invading pathogens and injurious self-stimuli.

A ‘resting’ or quiescent microglial cell has a very small cell soma with elongated ramified processes. The term surveillant is now considered to be a more accurate term to describe the state of a microglial cell in non-pathological conditions, as the microglia are not dormant, but are rather constantly monitoring and sampling the CNS environment. Under normal conditions, the radial processes are highly motile and are constantly extending and retracting, while the soma remains relatively stationary [3,4]. These processes do not overlap with the processes of adjacent cells, and the system is so fine-tuned that it is suggested that the entire brain volume is scanned by the microglia every few hours [3]. This surveying phenotype of microglia is maintained by a delicate balance between ‘stimulatory’ and ‘inhibitory’ signals. This includes numerous interactions between neurons and microglia. One such example is the interaction between CX3-chemokine ligand 1 (CX3CL-1, also known as fractalkine or neurotactin), which is produced by neurons, and its receptor CX3CR1, which is expressed on microglia [59]. CD200 is a surface molecule that is widely expressed on neurons, as well as astrocytes and oligodendrocytes [60]. Its receptor, CD200R, is found exclusively on microglia in the CNS and interaction between the two molecules also assists in keeping microglia in their quiescent state [61,62]. The quiescent state of microglia is not only regulated by exogenous signals, but also by endogenous transcription factors including runt-related transcription factor 1 (Runx1) and interferon regulatory factor 8 (Irf8) [63,64,65].

In response to an immune challenge, pathogen, or injury, these ramified surveilling microglial cells undergo an intricate, multi-stage activation process and their morphology is rapidly changed to an ‘activated’ microglial cell with an amoeboid form. These macrophage-like cells have retracted processes, are spherical in shape with an enlarged cell body, and contain phagocytic vacuoles [47,66]. Chemotactic reorientations of the microglial processes towards the pathological stimulus can occur within minutes. The processes form an area of containment around the site of injury, separating the injured tissue from the healthy tissue, suggesting that microglia may be the first line of defense following injury [4]. Activated microglia are virtually indistinguishable from macrophages not only in their morphology, but also with regards to their surface markers and function. Microglia-expressed macrophage-associated markers include CD11b, Iba1, and EGF-like module-containing mucin-like hormone receptor-like 1 (EMR1, also known as F4/80) [67]. Contributing further evidence of the similarity of microglia to macrophages, mice lacking the PU.1 transcription factor, which is a crucial transcription factor in the differentiation of many myeloid cells including macrophages, do not have microglia present in the CNS [68,69]. In recent years more specific markers of microglia have been identified, which allow microglia to be distinguished from infiltrating monocytes or CNS macrophages, including transmembrane protein 119 (TMEM 119) [70], and the purinergic G-protein coupled receptor P2RY12 [71]. However, microglia can shift activation states in a context-dependent manner causing them to lose their microglia-specific markers, rendering it more difficult to distinguish between infiltrating myeloid cells [72,73]. For example, t-SNE (t-distributed stochastic neighbor embedding) analysis identified clusters of microglia with low to negative levels of TMEM119 and P2RY12 from brain samples of patients with multiple sclerosis compared to homeostatic healthy controls that are enriched for those genes [73].

Due to the striking similarities in their morphologies, an early concept emerged to classify the microglial activation states in a similar manner as used for peripheral macrophages: the classically activated (M1-type) state and the alternatively activated (M2-type) state. This concept was based on the ability of a stimulus to induce cytokines that are either pro-inflammatory (M1) or anti-inflammatory (M2), and parallels the Th1 and Th2 terminology used for T cells. However, in more recent years, single-cell transcriptome studies have identified microglia signatures that overlap with both M1-like and M2-like profiles, suggesting they do not follow the same polarization model as peripheral macrophages [74,75]. Moreover, the M1/M2 polarization paradigm of microglia was helpful when conceptualizing the behavior of microglia in vitro, yet the phenotypic bias is rarely seen in vivo. To add to the complexity, microglial signatures are sensitive to regional distribution and specific to the local environment, which can change under different conditions such as during neurodegeneration or aging [76]. In an elegant study, McColl and colleagues described discrete microglia transcriptional profiles from purified microglia in the cortex/striatum, hippocampus, and cerebellum of young adult mice [72]. Enrichment analysis of these microglia revealed regional expression profiles that correlated with distinct biological processes. The hippocampal microglia genes were associated with energy production and the cerebellar and cortical microglia genes were associated with immunoregulatory pathways. Notably, aging resulted in non-uniform transcriptome changes across regions with aged, 22 month old hippocampal microglia suppressing genes involved in immune function such as cell-adhesion (*CD36* and *CD93*) and antigen processing (*MHC-II* genes *H2-Aa* and *H2-Ab1*) compared to microglia from young 4 month old mice.

Despite its common usage in the literature, this M1/M2 paradigm to describe the two contrary facets of the inflammatory response of microglia is no longer accurate. In reality, the microglial population in the brain is not all polarized to one extreme or the other at a particular point in time, but instead has varying transcriptional profiles dependent on the local microenvironment. With increasing technology and single-cell analysis, the field will need to consider new terminology to define microglial phenotypes, but currently the issue is unresolved.

### 2.3. Microglial Role in Acute Neuroinflammation

Classically, inflammation involves redness, swelling, heat, pain, and loss of function [77]. However, when referring to inflammation in the brain, this historical description is transcended as the inflammatory response is occurring on a cellular basis and therefore the classical definition is not relevant to the inflammatory processes occurring in the CNS. Neuroinflammation is thus simply characterized as an inflammatory response within the brain or spinal cord. Before neuroinflammation became part of the scientific vernacular, researchers used to term ‘reactive gliosis’ to describe the response of the CNS to injury, referring to the presence of enlarged glia cells in the vicinity immediately after the insult occurred. Since the first mention of neuroinflammation in a publication in 1995 [78], the number of papers on the topic on PubMed has exploded to over 17,000 as of 2019.

Activation of an inflammatory response after CNS injury, such as trauma, ischemia, or infection, is a necessary response to curtail injury and to repair damage. Acute neuroinflammation in retort to such an insult should be of a relatively short duration and graded in relation to the stimulus intensity, and should result in elimination of the offending cells or damage and initiation of a tissue repair response. Mediators such as chemokines, cytokines, and reactive oxygen species (ROS) produced by CNS cells transmit messages to manage this inflammatory response [79]. In normal conditions, the immune response is fine-tuned and balanced in order to achieve this goal, but in pathological conditions, the responses can become skewed. As the factors produced in the inflammatory response are themselves capable of inducing tissue damage, if this imbalance occurs they can upset the fragile homeostasis of the CNS and thus cause cell dysfunction or cell loss.

Both the innate and adaptive immune systems are involved in the inflammatory response, which involves an immense number of cells and factors. Microglia, as the intrinsic immune cells of the CNS, play an inherent role in neuroinflammation, as they perform the primary immune surveillance and defense functions of the CNS. Astrocytes, endothelial cells, and peripherally derived immune cells also contribute to the response. However, due to the crucial role microglial cells have, they are often the focal point of any discussion of neuroinflammation. They are constantly surveying the CNS, rapidly expanding and retracting their processes as they palpate their microenvironment [3]. They are also extremely sensitive to minute changes in the environment of the CNS, such as alterations in ion homeostasis and can become activated in response to such changes even before pathological damage is detectable [80]. Microglia have a unique set of membrane channels and it is thought that these channels, particularly the inwardly rectifying potassium channels, may be responsible for this responsiveness [81,82]. Pattern recognition receptors (PRRs) are found on the surface of microglia, and recognize pathogen-associated molecular pattern molecules (PAMPs) and damage-associated molecular pattern molecules (DAMPs). These PRRs, which include Toll-like receptors (TLRs) and Nod-like receptors (NLRs) among others, are crucial to the immune response, and are thought to be among the first responders to CNS injury (reviewed in [83,84]). In addition, microglial cells also express a number of receptors including those for ATP [85], the neurotransmitters acetylcholine and noradrenaline [86], and calcitonin gene-related peptide (CGRP) [87]. Activation of these receptors can rapidly induce large quantities of genes necessary for the appropriate immune response [87].

Once activated, microglia change their morphology and also alter the self-expression of numerous markers. Microglia turn on expression of the major histocompatibility complex class II (MHC class II), which is important in antigen presentation and activation of naïve T cells [88]. It has been proposed that the specialized CNS lymphatic system plays a role in waste clearance and drainage of macromolecules, immune cells and intra-cerebrally injected antigens into the cervical lymph nodes in the neck ([89,90,91], reviewed in [92]). Draining CNS-derived antigens initiates a T cell response to extravasate into the brain parenchyma. Migration out of the cerebrovasculature occurs following chemokine signaling from the endothelial cells, which is mediated by the interaction of leukocyte integrins such as leukocyte function-associated molecule 1 (LFA-1) and adhesion molecules such as intercellular adhesion molecule 1 (ICAM-1) and vascular cell adhesion protein 1 (VCAM-1) (reviewed in [93]). Once across the brain-blood barrier (BBB), T cells are restimulated by local antigen-presenting cells from antigens presented on MHC class II [88,94]. A host of interacting molecules engage between microglia and the antigen-specific T cells to perform multiple functions including the maintenance of tolerance toward self-proteins and prevention of auto-immunity. Early evidence highlights a role of microglia in the process of T cell migration and activation in the CNS. Following axonal injury, microglia acutely upregulate adhesion molecules such as thrombospondin and ICAM-1, which are known to be important components of a T cell mediated adaptive immune response [88,95]. The exact ability of the microglia to present to naïve T cells compared to other antigen-presenting cells in the CNS (e.g., perivascular macrophages and astrocytes [88]) is not fully known, although this evidence combined does suggest that the microglia play a role in the initiation of the adaptive immune response.

The activation of microglia in the CNS after acute injury such as spinal cord injury, ischemic stroke or traumatic brain injury has been intensely studied. In a mouse model of ischemic stroke, Hu et al. [96] found that local microglia, as well as newly recruited macrophages, assumed a protective phenotype in the early stages following ischemia, peaking at 5 days post-injury, and this seems to be protective of the neurons. However, this gradually transitions to be dominated by a cytotoxic phenotype, perhaps primed by the ischemic neurons. A similar pattern was also seen in a model of traumatic brain injury [97]. In another study, the ablation of proliferating microglial cells increased the size of the infarction following ischemic injury, suggesting that the proliferating cells have a neuroprotective role [98]. The microglial response to acute brain injuries can persist for many months. After experimental focal brain jury in rats, microgliosis was observed 3 months after injury, alongside increased synthesis of pro-inflammatory cytokines such as TNF-α and IL-1β [99]. In humans, elevated numbers of microglia were seen up to four years after head injury [100]. However, if the cytotoxic microglial response persists in this fashion for an extended period of time after acute injury, the inflammatory response may become destructive rather than beneficial.

### 2.4. Microglial Role in Chronic Neuroinflammation and Neurodegeneration

Neuroinflammation was first suggested to have a role in neurodegenerative disease as early as the beginning of the 20th century. In 1907, the same year Alzheimer published his seminal paper first describing the disease that was to become his namesake [101], Oskar Fischer described plaque formation in patients with senile dementia. Fischer later stated that the plaque deposition was a result of an inflammatory reaction, but could not find the morphological characteristics of an inflammatory reaction [102,103]. Thus, the idea of inflammation playing a role largely lost favor as it was a widespread belief at the time that the CNS was an immune-privileged site, separated from the immune system by the blood–brain barrier. The discovery of microglia as the resident immune cells of the brain led to a breakdown of this belief, and though it took several decades for a rebirth of research into the role of inflammatory responses in neurodegenerative disease, microglia are now known to be key mediators of these responses (reviewed in [104]).

In contrast to the essential response of acute inflammation, chronic inflammation is a long-lasting, frequently self-perpetuating response. Rather than having a protective role by eliminating pathogens or encouraging tissue repair, chronic inflammation is often detrimental and is associated with tissue damage and blood–brain barrier breakdown. Inflammation in the CNS has hence been aptly described as a double-edged sword [105]; while acute inflammatory reactions are beneficial and can limit damage and promote tissue repair, chronic inflammation can severely damage brain tissue. Microglia are rapidly activated following harmful stimulus exposure and will eliminate toxins and phagocytose dead or dying cells. Upon elimination of the stimulus, the microglia will gradually revert to their surveying phenotype. However, if there is continuous exposure to a stimulus, then a self-sustaining cycle of chronic inflammation can occur, which is detrimental to the neuronal population.

With age, there is a chronic progressive increase in the inflammatory status in a phenomenon known as ‘inflammaging’ [106]. There are a large number of pathological conditions, which are affected by the inflammatory status, including neurodegenerative diseases such as Alzheimer’s disease (AD) and PD, which are both age-related disorders. This inflammation is low-grade, chronic, occurs in the absence of infection, and is largely driven by endogenous signals. There is a new field of research known as ‘geroscience’, which studies the link between aging and age-related chronic diseases [107], and the underlying mechanisms, of which inflammation is a pivotal central aspect. This age-related chronic inflammation is associated with an increased level of pro-inflammatory cytokines and a decreased secretion of anti-inflammatory cytokines (reviewed in [108]).

The phenomenon of microglial priming also performs an influential role in neurodegenerative disease. Microglial priming is a heightened microglial response (relative to naïve cells) to a second stimulus. Microglia can be primed by a variety of stimuli, such as chronic stress [109], a transient peripheral infection [110], or chronic exposure to low levels of pathogens [111]. In animal models of neurodegenerative disease, a systemic inflammatory challenge has been shown to produce an exaggerated immune response in the CNS [112,113]. Olsen et al. [114] also demonstrated that viral neuroinflammatory priming in the substantia nigra significantly exacerbated α-synuclein aggregate-induced neuroinflammation and neurodegeneration. Another group likewise observed an enhanced neurodegenerative effect by combination of the H1N1 influenza virus and MPTP [115]. However, it is not yet known how or if the response differs between different neurodegenerative conditions or different priming agents.

Chronic neuroinflammation is now generally accepted to be an important component in the progression of neurodegenerative diseases. Microglia are activated in these conditions, and their role as scavengers to help clear the debris of the dying neurons is critical. However, when the microglia are activated chronically, they can release an excess of cytotoxic factors, which can further contribute to neuronal death, and thus a self-sustaining cycle of neuroinflammation and neurodegeneration is initiated [116]. The link between neuroinflammation, microglial activation, and neuronal death has been examined in numerous neurodegenerative diseases. AD is the most common neurodegenerative disease and is associated with progressive cognitive decline and memory loss. Pathologically, a major hallmark of this disease is the presence of extracellular plaque deposits of the β-amyloid (Aβ) peptide [117]. This peptide is implicated in the pathology of AD, both through direct toxicity to neurons but also through the recruitment and activation of microglia [118,119], indicating an important role for microglia in this disease. In fact, in one study carried out in the post-mortem brains of patients, microglial activation was seen to be correlated with disease progression [120]. Huntington’s disease is an autosomal dominant inherited neurodegenerative disorder, characterized by motor, cognitive, and mental impairments, and is caused by the expansion of a CAG trinucleotide repeat in the gene that encodes the huntingtin protein [121]. This causes a preferential loss of medium spiny neurons in the striatum [122]. Marked microgliosis has been seen in the post-mortem Huntington disease brain [123,124], as well as in mutant gene carriers even before symptoms present [125], indicating that persistent activation of these cells may be involved in this disease. Microglia and chronic inflammation are also implicated in the progression of PD, which is discussed extensively in the next section.

### 2.5. Role of Microglia in Parkinson’s Disease Pathology

PD is a chronic, progressive neurodegenerative disease and is the second most common neurodegenerative disorder. It was first observed by James Parkinson in 1817, who described tremors, postural instability, altered gait, and falls in what he called the ‘shaking palsy’ [126]. However, it was not until the 1860s when Charcot and Vulpian noted the presence of bradykinesia that it was distinguishable from other neurological conditions [127]. Today, it is recognized clinically by tremor at rest, bradykinesia, muscle rigidity, and postural instability, although a variety of non-motor symptoms may also be present, including depression and anxiety, constipation, dementia, and sleep disturbances [128]. The exact cause of this disease has yet to be fully elucidated, but it is thought that both genetic and environmental factors play a role. Various genes that cause a familial inherited form of the disease have been identified, for example, mutations in the α-synuclein gene [129] or the leucine rich repeated kinase 2 (*LRRK2*) gene [130], as they have several environmental entities that increase the risk of disease such as the pesticide rotenone and the herbicide paraquat [131]. The incidence of the disease is also known to be age-associated, affecting about 1% of people over 60 years of age [132].

Pathologically, the most prominent characteristic of PD is the slow and progressive degeneration of dopamine neurons in the substantia nigra. By the time the motor symptoms appear, up to 80% of striatal dopamine and 50% of the nigral neurons have already been lost [133,134]. Another defining pathological feature of PD is the presence of the proteinaceous inclusions known as Lewy bodies, first described by Friedrich Heinrich Lewy in 1912 [135]. α-synuclein, a filamentous protein, is the main component of these aggregates [136]. The role of these inclusions in PD is still not fully understood despite intensive research. However, various cellular mechanisms have been identified that are thought to contribute to the neuronal degeneration such as oxidative stress, excitotoxicity, mitochondrial dysfunction, abnormal protein handling, and neuroinflammation (reviewed in [137]).

The presence of inflammatory processes in post-mortem PD patients has been verified through the analysis of levels of pro-inflammatory mediators. The cytokine TNF-α has been seen to be increased in both the substantia nigra and the striatum post-mortem [138,139], which is also the case with interleukin-1β [140,141] and interferon-γ [142]. Enzymes associated with inflammation that are expressed by microglia were also elevated in the substantia nigra, such as the enzymes inducible nitric oxide synthase (iNOS) and cyclooxygenase-2 (COX-2) [143], providing further evidence of neuroinflammation playing a role in PD.

There is extensive evidence for microglial activation in PD, and in PD models, and it is now widely accepted that this microglial activation contributes to neuronal death in a self-perpetuating manner. The first evidence of microgliosis in PD came from McGeer and colleagues in 1988 who reported the existence of reactive microglial cells in the substantia nigra of PD patients post-mortem [144]. These were identified as microglia due to the presence of HLA-DR on the surface of the cells, which is a receptor belonging to the class major histocompatibility complex II (MHC class II). This was later confirmed by other groups using different microglial markers such as ICAM-1 or CD68 [145,146]. This microgliosis was also seen in patients that developed rapid-onset severe parkinsonism after intra-venous injection of 1-methyl-4-phenyl-1,2,3,6-tetrahydropyridine (MPTP) [147]. MPTP is a by-product in the synthesis of 1-methyl-4-phenyl-4-propionoxy-piperidine (MPPP), which is an analog of the opioid meperidine. MPTP is transformed to the toxic metabolite MPP+ by monoamine oxidase B in the brain and can interfere with complex I of mitochondria, affecting the electron transport chain. It is taken up by the dopamine uptake site, becoming concentrated in dopamine neurons and resulting in their degeneration, hence the extreme parkinsonian bradykinesia [148]. The microglia of these patients also contained the dark pigment neuromelanin, which is expressed in large quantities in the dopaminergic cells in the substantia nigra, providing evidence that the amoeboid microglia are clearing the dead and dying neurons. Although this research has contributed much to our knowledge of PD, the fact that these studies were all carried out post-mortem is a limitation, as therefore the role of microglia in the progression of the disease cannot be simply elucidated from these observations. However, a large scale genome-wide association study (GWAS) of patients with late-onset sporadic PD carried out by Hamza and colleagues identified a single nucleotide polymorphism (SNP) in the *HLA-DR* gene expressed on microglia, which was a genetic risk factor for the disease [149], providing evidence in living patients that microglia may contribute to the advancement of the disease. Under certain conditions, this mutation also has been associated with a shift towards a CD4^+^ pro-inflammatory T cell response, suggesting that T cells are involved in PD [150]. Since the discovery of this SNP by this research team in 2010, several other common genetic variants associated with an increased risk of PD have been identified in the *HLA*s corresponding to MHC class II [151,152,153].

The protein α-synuclein plays a crucial role in the pathogenesis of PD, with mutations or triplications in this gene shown to cause familial forms of the disease [129,154]. In PD, α-synuclein is overexpressed and is present in various formations, and accumulates in the form of amyloid fibrils into Lewy bodies [136,155]. Microglia should be the main cell clearing this toxic protein, but α-synuclein has been shown to alter microglial activity. Research regarding altering of microglial phagocytosis by α-synuclein includes some contradictory results, depending on the type of α-synuclein used and the cell model. α-synuclein with the A53T mutation was seen to promote a pro-inflammatory profile and impaired phagocytosis in BV2 microglia [156], while Roodveldt et al. saw increased phagocytosis with this variant, but impaired phagocytosis with the A30P and E46K forms in primary microglial cultures [157]. A study using α-synuclein knock-out mice showed microglia with a basally increased reactive phenotype, and an impaired phagocytic ability, suggesting that non-pathological levels of α-synuclein are important in preventing inflammation and promoting phagocytosis [158]. Furthermore, research has shown the disparate conformations of α-synuclein differentially impact microglial phagocytosis, with monomeric forms promoting phagocytosis and oligomeric forms inhibiting it [159]. Age is also an important element in the ability of microglia to phagocytose α-synuclein, with older cells being less efficient at clearing the toxin [160].

The first report of the ability of α-synuclein to alter the microglial inflammatory profile came in 2005 by Zhang and coworkers [161], who showed that microglia phagocytose oligomeric α-synuclein, which then leads to shifting of the microglia to a pro-inflammatory phenotype, with increased prostaglandin E2 and increased ROS production, leading to enhanced dopaminergic neuron toxicity. There are now numerous studies showing that α-synuclein can spur microglia towards a cytotoxic, pro-inflammatory phenotype, with increased production of cytokines such as TNF-α, IL-1β, and IL-6 [162,163,164], and increased levels of the enzymes COX-2 and iNOS [163,165]. Similarly with the phagocytic ability, there is contrasting evidence with regards to the different α-synuclein disease-related mutations and their effect on the release of pro-inflammatory cytokines. One study reported almost no pro-inflammatory effect for the A53T α-synuclein and a high effect for the A30P [157,166], while another found that the A53T form had the highest pro-inflammatory effect on microglia, followed by A30P [166].

In addition to the effect that different mutations in the α-synuclein gene have on microglial phenotypes, other genes associated with an increased risk of PD are also thought to contribute to the regulation of microglial function, such as *LRRK2*. This gene encodes for a kinase that interacts with actin-regulating proteins and regulates actin dynamics, which is important for the moving of the processes of surveilling microglial cells. LRRK2-knockdown BV2 microglial cells have been reported to be highly motile compared to control cells [167], and to have an altered morphology compared to controls after challenge with lipopolysaccharide (LPS) [168]. LRRK2 alters microglial motility through its effect on focal adhesive kinase (FAK), a key regulator in cell movement [167].

In all classical animal models of PD, some type of microgliosis is present. This includes toxic models such as 6-hydroxydopamine (6-OHDA) [169], rotenone [170,171], and MPTP [172,173], and also non-toxic models like the axotomized models [174]. There are also PD models that work by causing over-activation of the immune system in the brain, such as the LPS model and the poly I:C (polyinosinic:polycytidylic acid) model [175,176,177], highlighting the relevance microgliosis has on neuronal survival. However, neuronal death is not strictly necessary for microglial activation. In transgenic mice overexpressing α-synuclein with the double mutation of A53T and A30P under the TH promotor, microgliosis occurred very early, long preceding cell death [165].

It is now clear that microglia and inflammation are important to the progression of PD. However, still relatively little is known about the microglial phenotypes with relation to each stage of the disease progression, thereby hindering the development of therapies to shift microglial activation states. Further studies of PD pathology are required to allow advantageous manipulation of microglial phenotypes, depending on how far the disease has advanced in each individual patient.

## 3. The Cannabinoid System

The endocannabinoid system is a relatively newly discovered system in neuropharmacology. However, research into this field has grown exponentially in recent decades and it is now recognized that the endocannabinoid system plays important physiological and pathological roles in neuroinflammation and neurodegeneration, alongside numerous other functions such as in pain, mood, and appetite.

### 3.1. Overview of the Endocannabinoid System

The term ‘cannabinoid’ refers to any compound whose effects are mediated through modulation of components of the endocannabinoid system, such as modulation of the endocannabinoid receptors, enzymes, or transport proteins. This term includes molecules found in the two main plant subspecies, *Cannabis sativa* and *Cannabis indica*, as well as endogenous and synthetic compounds. The potential of cannabinoids as therapeutic agents has been acknowledged from as early as the third millennium B.C. in China, where texts have been discovered that observe the beneficial effects of cannabinoids or cannabis in relief of rheumatic pain [178]. During the course of the subsequent millennia, cannabinoids remained in use both medicinally and recreationally. To date, there are over 100 cannabinoids that have been isolated from cannabis, including cannabidiol (CBD) identified in 1963 [179] and the main psychoactive component, Δ9-tetrahydrocannabinol (THC) identified in 1965 by Mechoulam and Gaoni, which constituted the first major breakthroughs in modern times [180]. It would be over two decades before the first THC binding site was discovered [181], but since then research into this field has grown expeditiously.

The effects of cannabinoids are primarily mediated through two major receptors type 1 and type 2, CB_1_ and CB_2_, respectively_._ The receptor now known as the CB_1_ receptor was first identified in 1990 [181] and in recent years the structure has been further elucidated [182,183]. This receptor is widely distributed in the central nervous system, particularly in the cerebellum, cortex, basal ganglia, and hippocampus [184,185]. In the CNS, it has been shown to be predominantly expressed on axons and terminals of neurons. CB_1_ receptors are also present at lower but still functionally relevant levels in many peripheral tissues and organs, including adipose tissue [186], liver [187], pancreas [188], and skeletal muscle [189].

Cloning of the CB_2_ receptor came in 1993, three years following the identification of the CB_1_ receptor [190] and the crystal structure of the receptor has subsequently been reported at a high resolution [191]. Originally, CB_2_ was informally referred to as the ‘peripheral receptor’ as it was found largely in immune tissues with highest mRNA levels in the tonsils and spleen and in peripheral immune cells and it did not appear to be present in the brain [192]. However, it is now widely accepted that this receptor upregulates its expression on activated microglia in the central nervous system [193,194]. The presence of the CB_2_ receptor on neurons in the CNS remained a debate for years with much contrasting evidence being published. It is now thought that the receptor is expressed on particular subsets of neurons at functionally relevant levels, although the different techniques used to identify CB_2_ expression can show varying results (reviewed in [195]). The human, mouse, and rat protein sequences of the CB_2_ receptor have been found to differ quite substantially in the C-terminus, with the mouse sequence being 13 amino acids shorter and the rat sequence 50 amino acids longer compared to the human protein [196]. Therefore, caution must be taken in extrapolating the results from non-human models to the effects of CB_2_ activation in humans. Furthermore, one of the greatest challenges currently in the field is the lack of reliable and specific antibodies against CB_2_, which has contributed to the conflicting reports on CB_2_ receptor distribution.

Both CB_1_ and CB_2_ receptors belong to the G_i/o_ family of seven transmembrane G protein-coupled receptors, although the identity between the two receptors in humans is remarkably low, with only 44% homology overall and 68% in the transmembrane helices, which contain the putative binding sites for cannabinoids [197]. Both receptors inhibit the enzyme adenylyl cyclase, resulting in a reduced synthesis of cyclic AMP, and activate the mitogen-activated protein kinase pathway by signaling through G_i/o_ proteins. Furthermore, CB_1_ activation can modulate certain voltage-gated calcium channels and inwardly rectifying potassium currents [198]. The CB_2_ receptor also affects additional pathways, such as activation of phospholipase C, leading to calcium release [199], and activation of the phosphatidylinositol 3-kinase pathway [200].

Equipped with the rationale that receptors would not be present in the body without the existence of an endogenous ligand, following the discovery of the cannabinoid receptors researchers began the search for these ‘endocannabinoids’. The two best characterized are arachidonoyl ethanolamide (usually referred to as anandamide or AEA) and 2-arachidonoyl glycerol (2-AG), although other endogenous molecules have also been discovered that either interact in some way with the endocannabinoid system (e.g., 2-arachidonyl glyceryl ether (noladin ether) [201]), or endocannabinoid-like lipids that do not act via cannabinoid receptors (e.g., palmitoylethanolamide (PEA) and oleoylethanolamide (OEA) [202,203]). Unlike most classical neurotransmitters, anandamide and 2-AG are not stored in vesicles, but are synthesized ‘on demand’ (usually by membrane depolarization or activation of certain G-protein coupled receptors) from their lipid membrane precursors. Upon their postsynaptic release they act as retrograde messengers, suppressing transmitter release from neurons in either a transient or long-term manner, roles that have been well established [204,205,206]. These two endocannabinoids possess distinct properties with regards to their interactions with the cannabinoid receptors. 2-AG works as a full agonist at both receptors with low-to-moderate affinity. Anandamide, on the other hand, has very low activity at the CB_2_ receptor but is a high-affinity, partial agonist at the CB_1_ receptor [207]. Anandamide also has affinity at transient receptor potential vanilloid type 1 (TPRV1) channels [208], although the strength of its agonism seems to be strongly dependent on the level of receptor reserve in the tissue [209,210]. Besides the aforementioned receptors, endocannabinoids and phytocannabinoids also interact with the orphan receptor G protein-coupled receptor 55 (GPR55) [211] and peroxisome proliferator-activated receptors (PPARs) [212,213].

As well as having disparate intrinsic efficacies, anandamide and 2-AG also have distinct biosynthesis and metabolism pathways. Anandamide is primarily formed through cleavage of the phospholipid *N*-arachidonoyl-phosphatidylethanolamine (NAPE) [214] by a specific phosphodiesterase enzyme known as *N*-acyl phosphatidylethanolamine-specific phospholipase D or NAPE-PLD [215]. Ultimately, the inactivation of anandamide is carried out by an enzyme named fatty acid amide hydrolase (FAAH) through hydrolysis of the amide bond to form ethanolamine and arachidonic acid [214]. 2-AG is a monoacylglycerol and is formed through the hydrolysis of diacylglycerols (DAGs) by diacylglycerol lipases (DAGLs) α and β, with the two isoforms differentially expressed in developing and adult nervous tissue [216]. 2-AG can be metabolized by several different chemical reactions. It is mainly degraded by monoacylglycerol lipase (MAGL) to arachidonate and glycerol, although the αβ-hydrolases ABHD6 and ABHD12 can also catalyze its hydrolysis [217].

Many synthetic cannabinoid compounds have now been developed, including specific agonists, antagonists, and inverse agonists, which have different affinities and efficacies at the cannabinoid receptors. Molecules that act on enzymes or transporter proteins involved in the cannabinoid system have also been generated. The creation of these molecules and the isolation of the vast array of cannabinoid compounds found naturally in the *Cannabis* plants, allows researchers to better investigate the physiological functions of the cannabinoid system, and thus advance potential therapies for neurological disorders. For the structures and pharmacological profiles of the cannabinoids mentioned throughout the review, see the comprehensive review by Pertwee and colleagues [212].

### 3.2. The Cannabinoid System in Inflammation and Immune Modulation

Mounting evidence indicates that the cannabinoid system has a major function in the modulation of the immune response and inflammation, both centrally and peripherally. Therefore, this system has the potential to be manipulated in order to provide therapeutic effects in diseases with an inflammatory component.

The presence of both the CB_1_ receptor and the CB_2_ receptor on immune cells was one of the first pieces of evidence to indicate that the endocannabinoid system might play a role in the immune response [192]. Results from subsequent in vitro and in vivo studies suggest that cannabinoids execute their immunomodulatory effects in numerous ways: by induction of apoptosis, by suppression of cell proliferation, by modulation of immune cell migration, by increased anti-inflammatory cytokine production and inhibited production of pro-inflammatory cytokines and chemokines, and by modulation of the expansion of regulatory T cells [218,219]. Cannabinoid compounds have been seen to cause alterations in immune function from as early as the 1980s, a decade before the cannabinoid receptors were even characterized. Tindall et al. [220] detected a more rapid progression from HIV infection to AIDS in marijuana smokers compared to those who did not use the drug. HIV-positive individuals who use marijuana also had an increased risk of bacterial pneumonia, opportunistic infections, and Kaposi’s sarcoma [221,222]. Alveolar macrophages obtained from the lungs of habitual marijuana smokers who were otherwise healthy individuals showed a decreased phagocytic ability, decreased cytotoxicity, and decreased cytokine production [223]. Clearly, exogenous cannabinoids affect the immune system and if this effect could be manipulated, it could be beneficial in the treatment of a vast number of conditions.

As stated in the previous section, in the brain, CB_1_ receptors are predominantly found on the terminals of neurons, where they play a role in neurotransmitter release. However, as they are also present on immune cells, albeit in relatively low quantities, ergo they can have an effect on immune modulation. mRNA analysis showed that with regards to human peripheral immune cells, the highest levels of CB_1_ expression were seen in B cells, followed by natural killer cells, and with varying expression in several other blood cell types including monocytes and lymphocytes [192]. Multiple sources of evidence suggest that the CB_1_ receptor on immune cells could be a potential target for the regulation of inflammation. Much evidence exists for a role of the CB_1_ receptor in the chronic demyelinating disease multiple sclerosis (MS), which is an immune-mediated disease involving the demyelination of neurons by CD4^+^ T cells. In post-mortem brain tissue from MS patients, CB_1_ staining co-localized with CD68^+^ macrophages and CD3^+^ T cells in areas of active lesions (i.e., areas with activated microglia) [224]. As expected, this study also reported CB_1_ staining in MAP2^+^ neurons and MBP^+^ oligodendrocyte cells. Animal models of MS such as the experimental autoimmune encephalomyelitis (EAE) model found immune modulation or disease amelioration through CB_1_ receptor agonism [225,226,227,228]. Furthermore, anandamide, through a CB_1_-dependent mechanism, inhibited Theiler’s virus-induced vascular cell adhesion molecule-1 (VCAM-1) expression in mice, a receptor that is involved in leukocyte transmigration across the blood–brain barrier, which contributes to the pathology in MS [229].

Apart from these immunomodulatory effects, CB_1_ can also have beneficial roles in neuroprotection by the inhibition of excitotoxicity. A number of observations suggest that excitotoxicity contributes to the pathology of MS [230,231,232]. The CB_1_ receptor can be found presynaptically and can modulate glutamate release [233], and thus has a critical role for excitotoxicity control in neurological conditions. However, despite the immunomodulatory and anti-excitotoxic effects associated with targeting the CB_1_ receptor, research has largely focused on the CB_2_ receptor as a potential target in the endocannabinoid system to modulate the immune response and inflammation. This is based on the undesired psychoactive effects of CB_1_ activation, and the associated safety concerns. In addition, the therapeutic window to target the CB_1_ receptor following acute injury is likely to be relatively short, as most neuroprotective drugs in response to acute brain injury need to be administered within 6 h of injury onset [234]. As the CB_2_ receptor is thought to be devoid of psychoactivity due to its restricted presence on CNS neurons, and as it is expressed at vastly greater numbers on immune cells and tissues than the CB_1_ receptor, the CB_2_ receptor has been the focus of much research investigating its potential as a therapeutic immunomodulatory or anti-inflammatory target.

CB_2_ receptors are expressed in high levels in peripheral immune tissues, such as the spleen and the tonsils, and at levels in immune cells, which are 10–100 times the levels of expression of the CB_1_ receptor. The immune cells express the CB_2_ receptor to different extents, with the rank order being B cells > natural killer cells > monocytes > neutrophils > CD8 lymphocytes > CD4 lymphocytes [192]. Dendritic cells have also been shown to express CB_2_ receptors [235]. However, the level of expression of this receptor on immune cells is dependent on both the activation state of the cell and the nature of the activating stimulus. Lee et al. [236] found that stimulation with LPS substantially downregulated CB_2_ mRNA expression in splenocyte cultures in a dose-dependent manner. Contrastingly, stimulation with antibodies against cluster of differentiation 40 (CD40), which is a costimulatory molecule expressed by antigen-presenting cells, upregulated CB_2_ expression. This CD40-mediated upregulation was also seen in peripheral blood and tonsillar B cells [237]. CB_2_ upregulation has also been reported in response to IFN-γ in both microglia and macrophages [193].

As previously mentioned, one way cannabinoids execute their immunomodulatory role is by affecting the apoptosis of immune cells. As early as 1994 Schwarz and colleagues [238] demonstrated that high concentrations of THC or anandamide could induce apoptosis of B and T lymphocytes. Zhu et al. [239] later demonstrated that THC induced apoptosis in both lymphocytes and macrophages, and that Bcl-2 and caspase-1 were involved. It was noted that fragmentation preceded membrane damage, suggesting that THC was inducing apoptosis rather than necrosis of cells. This THC-induced immune suppression via T cell apoptosis was later exhibited in vivo in C57BL/6 mice [240]. Use of CB_2_ antagonists, but not CB_1_ antagonists, blocked apoptosis in these cells, indicating that THC induced apoptosis in a CB_2_ receptor-dependent manner. This suggests that targeting the CB_2_ receptor may be a promising approach to treating inflammatory and autoimmune diseases. Evidence to confirm this included the use of the synthetic CB_2_ agonist, JWH015, which in a dose-dependent manner both inhibited proliferation and induced apoptosis of splenocytes and thymocytes [241]. Cannabinoids are also seen to induce the apoptosis of malignant immune cells [242,243,244], insinuating that CB_2_ receptor activation could also be a novel therapeutic modality against immune system malignancies such as lymphomas and leukemias.

In addition to affecting apoptosis, cannabinoids also affect the proliferation of immune cells. Anandamide has been seen to inhibit mitogen-induced proliferation of B and T lymphocytes, at concentrations relevant to the regulation of neuronal responses. Δ8-tetrahydrocannabinol and the non-selective cannabinoid agonist CP55,940 also inhibited lymphocyte proliferation, but to a lesser extent [238]. 2-AG was observed to have an effect on the proliferation of mouse splenocytes in culture, an effect that seems to be dependent in part on cell density [245]. Cannabinoids have been seen to have a biphasic role with regards to both B cells and T cells. In B cells, increased proliferation was demonstrated in response to THC at low nanomolar concentrations [246], whereas another study showed that THC caused a reduction in LPS-induced proliferation of B cells [247]. A similar effect was seen in T cells, with high doses of THC being inhibitory and low doses being stimulatory [248]. This biphasic response should be taken into consideration when examining the effects of cannabinoids on immune cell proliferation.

Cannabinoid receptor activation has been shown to modulate the migration of both central and peripheral immune cells, which is an important element to acknowledge when studying diseases with an inflammatory component. The synthetic cannabinoid CP55,940 decreased the in vitro migration of rat macrophages, an effect that was attributed to both cannabinoid receptors, although CB_2_ had a more substantial effect [249]. In addition to affecting macrophage migration, the CB_2_ receptor also regulates the migration of neutrophils, NK cells, T cells, and B cells, with different agonists seen to cause varying effects. Endocannabinoids, phytocannabinoids, and synthetic cannabinoids can differentially modulate second messenger pathways in a phenomenon known as ‘agonist trafficking’ [250]. This phenomenon is thought to be relevant for CB_2_-induced cell migration. To use the case of T lymphocytes as an example, several laboratories have found that activation of CB_2_ receptors inhibits T cell migration in response to the chemokine CXCL12, with different ligands inhibiting migration to different extents. One group reported that anandamide and the CB_2_ agonist JWH133 reduced the migration of T lymphocytes, with the CB_1_ agonist docosatetraenylethanolamide (DEA) having no effect [251]. Another study reported that the non-selective agonists WIN55,212-2 and CP55,940 inhibited the chemotaxis of both CD4^+^ and CD8^+^ primary T-lymphocytes, as well as Jurkat cells. The CB_2_ agonist JWH015 also elicited this response, but high concentrations were required (10–40 μm) and the CB_2_ inverse agonist AM630 only partially reserved this effect, suggesting that other receptors are involved [252]. Clearly, activation of the CB_2_ receptor has a role in the modulation of immune cells but undoubtedly further studies are required in order to make targeting this process a feasible therapeutic approach (reviewed in [253]).

T helper cells are important enforcers of cell-mediated (Th1) and humoral (Th2) adaptive immunity. Cannabinoids have been demonstrated to regulate the balance of T helper 1 (Th1) and T helper 2 (Th2) cytokines in murine studies, with a downregulation of Th1-associated cytokines such as IFN-γ, IL-2, and IL-12, and an increase in levels of Th2-associated cytokines such as IL-4, IL-10, and TGF-β [254,255,256,257]. These effects are thought to be modulated to a considerable extent by the CB_2_ receptor, as evidenced by blockade of the majority of these effects by the CB_2_ receptor antagonist, SR144528 [254,258]. Th1 cytokines have been implicated in the pathogenesis in a number of conditions, including MS [259], rheumatoid arthritis [260], and primary sclerosing cholangitis [261]. Suppression of Th1 responses has been effective in inhibition in animal models of inflammatory disease such as rheumatoid arthritis [262,263] and EAE [264,265], suggesting cannabinoid manipulation of this response could be a helpful therapeutic agent for inflammatory disease.

Cannabinoids are well established as modulators of the immune system, affecting a variety of immune functions in humans and animals. With further research, this might be exploited in future therapies for numerous disorders, such as rheumatic disease, atherosclerosis, allergic asthma, and neurodegenerative diseases.

### 3.3. The Cannabinoid System, Microglia, and Microglia Phenotypes

As is the case with immune cells in the periphery, the activity of microglia can be modified by cannabinoids. There is evidence that microglia possess a complete endocannabinoid system, and that the expression or production of some of the components of this system are altered in neuropathological states.

In the CNS, endocannabinoids are produced by both neurons and glial cells such as microglia [204,266]. Microglial cells in culture produce both 2-AG and anandamide, with calcium ionophores and ATP selectively and substantially increasing 2-AG production [267,268]. This ATP-induced increase in 2-AG production has been shown to be due to the activation of P2X purinoceptor 7 (P2X7) ionotropic receptors, which are highly permeable to calcium. The subsequent sustained increase in intracellular calcium induced by the activation of these receptors increases DAGL activity, the enzyme that produces 2-AG, while inhibiting MAGL activity, the enzyme that degrades 2-AG [269]. It is suggested that microglia may be the main producers of endocannabinoids under neuroinflammatory conditions. This is supported by studies that show that microglia produce approximately 20-fold more endocannabinoids than neurons and astrocytes in culture [268,270]. In addition, 2-AG production is significantly diminished in P2X7 knockout mice in an EAE model [271]. As this receptor is only expressed by activated microglia, this supports the hypothesis that the synthesis of endocannabinoids is closely linked with the microglial activation state. Mecha et al. [272] found that the different in vitro microglial phenotypes were associated with an altered synthesis of endocannabinoids, with IL-4 and IL-13-stimulated microglia (the regeneration and repair subtype) selectively producing 2-AG, and TGF-β-stimulated microglia (the acquired-deactivation subtype) producing AEA.

Microglia in culture also express enzymes of endocannabinoid biosynthesis and degradation such as FAAH and MAGL, which can similarly be manipulated by microglial activation states [269,273]. Primary microglia stimulated with IL-4 and IL-13 were shown to induce a time-dependent rise in *DAGLα* gene expression while TGF-β stimulation induced the accumulation of NAPE-PLD and a reduction in FAAH mRNA, consequently activating anandamide and 2-AG, respectively. The activity of the serine hydrolase ABHD6 is also important for the regulation or inactivation of cannabinoids in the BV2 microglial cell line [217,274]. The existence of this novel enzyme expressed by microglia is promising with regard to a means of enhancement of endocannabinoid signaling, and selective inhibition of cannabinoid-degrading enzymes could be used as a potential therapy in neuroinflammation. Recognizing that microglial activation states are more complex in vivo, it is clear more studies are necessary to understand the role of endocannabinoids in microglial function.

In addition to producing endocannabinoids, microglia also express functional cannabinoid receptors. As is the case for macrophages and other peripheral immune cells, the expression of the cannabinoid receptors is related to their activation state. It is thought that in the healthy brain, resting microglia do not express the CB_2_ receptor, as the mRNA encoding for these receptors is undetectable or only detectable in trace amounts [190,275,276]. This is contrary to microglial cells in primary culture, which seem to be intrinsically ‘primed’, probably because of the methods used to transfer them into culture [277]. Numerous laboratories have shown that these ‘primed’ microglia prepared from murine or human tissue express CB_2_ receptors [193,278,279]. The expression levels of the receptors seem to vary depending on stimulus exposure, with LPS inducing a downregulation in both the CB_1_ and CB_2_ receptor on microglia, and with IL-4 and IL-13 or TGF-β inducing an upregulation in the two receptors [272]. However, it is generally accepted that a massive upregulation of the CB_2_ receptor on microglia occurs in response to inflammation or injury, as evidenced by animal models of a variety of diseases and species, from simian immunodeficiency virus-induced encephalitis in macaques, to stroke in mice, and paclitaxel-induced neuropathy in rats [194,280,281,282,283]. In vivo, the phenotype of the microglia and the upregulation of CB_2_ receptors have been shown to vary depending on the neuropathology or type of insult. For example, an increase in CB_2_ receptor expression and microglial activation was seen in the rat spinal cord in a chronic model of neuropathic pain, but not a peripheral inflammatory pain model [284].

The CB_1_ receptor is controversial with regard to the effect of its activation on microglia. This receptor has been reported in microglial cultures from mice, rats, and mollusks, but not humans. Different consequences have been seen after CB_1_ activation in different species, with increased nitric oxide production seen in mollusks but decreased production in rats [285,286]. Therefore, the CB_1_ receptor is not a major focus of interest for researchers examining the potential of cannabinoids on microglial modulation.

Multiple lines of evidence demonstrate the role of cannabinoids to regulate microglial cytokine production (Table 1). Early reports that involved antagonism of the CB_2_ receptors on microglia in culture were shown to increase the mRNA levels of pro-inflammatory cytokines such as IL-1α, IL-6, and TNF-α, suggesting that agonism of these receptors would induce a reduction in pro-inflammatory cytokines [287]. Follow-up studies confirmed that either the non-selective CB receptor agonist, CP55,940, or the CB_2_ selective agonist JWH015, lead to a dose-dependent reduction in LPS-induced production of TNF from rat primary cortical microglia [288,289]. Similar blockade of microglial TNF production following fibrillar Aβ incubation was shown after treatment with non-selective CB receptor agonist HU-210 or WIN55,212-2 and with CB_2_ selective agonist JWH133 [290]. More recently, reports have highlighted the benefit of cannabinoid signaling to increase microglia anti-inflammatory cytokine signaling. Correa et al. demonstrated that microglia isolated from the forebrain of neonate mice and treated with either JWH133, a CB_2_ receptor selective agonist, or AEA, further enhanced the LPS/IFNγ-induced expression of IL-10 [291]. Similarly, 2-AG and AEA increased primary rat microglia expression of Arg-1 towards an in vitro protective phenotype, suggesting endocannabinoids can regulate microglia by amplifying the wound healing profile and restraining the pro-inflammatory effects of microglia [272]. The anti-inflammatory response of Arg1 was shown to be mediated by CB_2_ receptors as demonstrated by reduced Arg1 expression in IL-4 and IL-13-stimulated microglia from CB_2_ receptor deficient mice. Interestingly another class of drugs known as CB_2_ receptor inverse agonists, such as SMM-189, have demonstrated similar reductions in pro-inflammatory cytokines from stimulated human microglia as seen from CB_2_ agonists [292]. The increased phosphorylation and translocation of CREB have been proposed as the cellular pathways that promote the CB_2_ inverse agonist anti-inflammatory effects, suggesting another avenue for the use of cannabinoids to regulate microglial inflammation.

Cannabinoids signaling also has shown a role in the functional behavior of microglia including both their phagocytic and migratory activity (Table 1). Primary microglia deficient in CB_2_ receptor expression engulfed significantly fewer fluorescent microspheres than the wild-type microglia following IL-4 and IL-13 stimulation, suggesting the impact of CB_2_ receptors on the phagocytic function of microglia [272]. As well as affecting chemotaxis of peripheral immune cells, cannabinoid receptors have also been reported to modulate microglial migration. 2-AG was found to be a very efficacious ligand regarding this response on the BV2 mouse microglial cell line, an effect that was prevented by cannabinol and cannabidiol, by blocking CB_2_ and cannabidiol-sensitive receptors respectively [268]. Another study corroborated that 2-AG-induced BV2 migration relies on CB_2_ receptors as demonstrated by the blockade of microglia recruitment after treatment with the CB_2_ inverse agonist SR144528 [293]. Anandamide increased BV2 cell migration in a concentration-dependent manner, as did the two putative endocannabinoids homo-γ-linolenylethanolamide (HEA) and docosatetraenylethanolamide (DEA), whereas another putative endocannabinoid, palmitoylethanolamide (PEA), which does not act on CB_1_ or CB_2_ receptors, had no effect [268]. However, immortalized BV2 cells have shown to produce different migration and cytokine responses than primary microglia, including higher migratory rates in immortalized microglia cell lines compared to primary cells [294]. To that point, rat primary microglia treated with a CB_2_ receptor agonist, JWH015, decreased LPS-induced chemotaxis, which is incongruent with cell line data given the same reduced migratory response was found from BV2 cells when CB_2_ receptors were blocked instead of activated [289].

Due to all the modulatory effects cannabinoids have on microglia, in addition to the massive upregulation of the CB_2_ receptor on microglia in neuroinflammatory states, there is an acute interest in harnessing these immune-modulatory effects for therapy in neurodegenerative diseases. Yet, as the varying responses and behaviors of the different microglia preparations (cell lines vs. primary) questions their translation in vivo, more research is needed to understand cannabinoid specific immune-related functions and how they act on neuroinflammation in model systems.

### 3.4. The Cannabinoid System in Neuroinflammation and Neurodegeneration

In 2003, one of the first pieces of evidence that alterations in the endocannabinoid system relevant to neuroinflammation occur in neurodegenerative disease came from a study by Benito and colleagues [295]. They found that in the hippocampus and entorhinal cortex of AD patients, there was a substantial and specific overexpression of the CB_2_ receptor on microglia in the neuritic plaques, while CB_1_ receptor expression was not altered. This upregulation of CB_2_ on microglia surrounding senile plaques has been confirmed with additional studies [296]. One study found a correlation between expression levels of the CB_2_ receptor and senile plaque score and Aβ(42) levels, which are two major pathological molecular markers of AD [296]. In addition to an upregulation in AD, a substantial increase in CB_2_ receptor expression has been found in human CNS tissue in a number of disorders associated with neuroinflammation, including MS, Down syndrome, and amyotrophic lateral sclerosis (ALS) [224,297,298]. It has been suggested that the upregulation and activation of the CB_2_ receptor in neurodegenerative disease may be part of a type of negative-feedback loop in response to physiological stress, with the aim of limiting the inflammatory process (reviewed in [299]).

Alterations in expression levels of enzymes in the endocannabinoid system have also been observed in neurodegenerative disease. In AD, increased expression of FAAH, the primary enzyme responsible for the metabolism of anandamide, was noted in astrocytes associated with senile plaques [295]. Anandamide is converted to arachidonic acid by FAAH, and the abundance of this enzyme in astrocytes in this disease suggests that astrocytes could be a major source of arachidonic acid and related pro-inflammatory compounds in the vicinity of these plaques.

Activation of the CB_2_ receptor has been shown to have anti-inflammatory effects in various animal models of acute and chronic neuronal diseases in which inflammation is involved, including stroke, traumatic brain injury, MS, and AD. In the case of stroke and traumatic brain injury, many different categories of cannabinoids were demonstrated to have neuroprotective effects, including non-selective cannabinoids, CB_2_-selective compounds, and cannabinoids without activity at the classical cannabinoid receptors [292,300,301,302,303,304]. However, some of the studies researching the potential of cannabinoid compounds for acute brain injury have been conducted with administration of the cannabinoid before injury, which is a scenario that is not possible for treatment of patients, and therefore the results of these studies and their translatability to potential therapies should be approached with caution. The number of clinical trials with cannabinoids in these diseases is limited, with one of the most relevant being a multicenter placebo-controlled phase III trial investigating dexanabinol in traumatic brain injury [305]. Dexanabinol is a synthetic cannabinoid derivative that does not have cannabinoid activity but instead acts as an NMDA antagonist. Despite its promising pre-clinical effects [300,301,306], the trial found it was safe but not efficacious in traumatic brain injury.

Due to the slow progression of chronic neurodegenerative diseases, there is a greater opportunity for therapeutic intervention with cannabinoids compared to acute brain injury conditions. With regards to AD, cannabinoids, including the CB_2_ selective agonist JWH133, were demonstrated to block Aβ peptide-induced activation of microglia in vitro, including a reduction in the release of the pro-inflammatory cytokine TNF-α, which is associated with the cytotoxic microglial phenotype, and a reduction in mitochondrial activity [290]. In co-cultures with neurons, the cannabinoids also prevented microglial-mediated neurotoxicity after Aβ exposure. In a transgenic Tg2576 mouse model of AD, which overexpresses a mutant form of amyloid precursor protein (APP), WIN55,212-2 and JWH133 both reduced the increase in TNF-α and Aβ levels. In addition, JWH133 reduced cognitive defects as measured by the novel object recognition test, and decreased microglial cell density [307]. In the Theiler’s murine encephalomyelitis virus-induced demyelinating disease (TMEV-IDD) model of MS, treatment with WIN 55,212–2, ACEA (a CB_1_ agonist), or JWH-015 (a CB_2_ agonist) showed a marked reduction in microglial activation visible in their morphology, as well as functional recovery in the rotarod test [227]. In the EAE model, administration of 2-AG ameliorated acute and chronic phases of the disease, accompanied by a polarization of macrophages towards a non-cytotoxic, protective phenotype [308]. These beneficial effects of CB_2_ activation are due to their presence on microglia, and are associated with suppression in microglial activation [234,309], and hence an inhibition of the release of cytotoxic factors that cause neuronal damage.

The potential therapeutic effects of CB_2_ specific agonism in neurodegenerative diseases are of great interest, due to the considerable limitations of current treatments. Further research is required, but in the future CB_2_ agonists could be administered to reduce the pro-inflammatory cytotoxic effects in diseases with a neuroinflammatory component for therapeutic gain.

## 4. Cannabinoids in Parkinson’s Disease: Therapeutic Implications of Targeting Microglia

As previously stated, in recent years it has become apparent that in neurodegenerative disease there is a self-sustaining cycle of neuroinflammation and neuronal death, with dying neurons activating microglia, which then can release factors that cause further neuronal death (reviewed in [310,311]). The concept of pharmacologically targeting the cannabinoid system in PD is based on the upregulation of cannabinoid receptors in PD patients identified in both the substantia nigra and the hippocampus, providing a neuroanatomical basis [312,313]. The in vitro evidence supporting the anti-inflammatory regulation of cannabinoid receptors on microglia suggests that cannabinoids may have the potential to benefit microglia dysfunction in PD, and thus slow or even prevent the dopaminergic degeneration.

Although this review is focused on the role of cannabinoids in neuroinflammation, when discussing the cannabinoid system in the context of PD, it is important to note that the CB_1_ receptor is highly expressed in the basal ganglia [314,315], indicating they may have a role in motor control. Although density is highest in the substantia nigra, it is not the dopaminergic nigrostriatal neurons that express the receptor. Instead, it is the medium spiny γ-aminobutyric acid (GABAergic) neurons that project to the substantia nigra from the striatum that are responsible for this high expression [316]. These neurons co-express the dopaminergic D_1_ and D_2_ receptors with CB_1_ receptors in the striatum [317]. CB_1_ knockout mice have more severe motor deterioration and neurodegeneration, as well as a reduced severity of L-DOPA-induced dyskinesias [318], highlighting the importance this receptor has in motor control and its link to the dopaminergic system. Researchers are investigating the potential use of cannabinoids for therapy in PD from a number of aspects: for alleviation of motor symptoms, alleviation of drug-induced side effects, and disease-modifying effects such as effects on α-synucleinopathy and through direct neuroprotection. Indeed, a number of clinical trials have been carried out examining the therapeutic potential of cannabinoids with regards to these aspects of PD (reviewed in [319]). However, these facets are outside the scope of this review.

The first indication that the endocannabinoid system may have a role in neuroinflammation in PD came in 2005 when Lastres-Becker and coworkers [320] exposed the cannabinoid agonist, HU-210 to a cerebrallar granule cell culture, which was treated with 6-OHDA (Table 2). HU-210 increased cell survival when the cells were directly exposed, even more so when the neuronal cultures were exposed to conditioned media from mixed glial cell cultures that had been treated with HU-210. This suggests that the drug exerted its neuroprotective effect largely by regulating the glial influence on neurons. Of note, the mixed glia cultures were comprised of 70% astrocytes and 30% microglia, yet highlighting the desirable effects of cannabinoids on glia in general. Further studies by another group using the non-selective agonists WIN55,212-2 and HU-210 in the MPTP mouse and the LPS rat, demonstrated profound anti-inflammatory effects, with reduced CD11b^+^ microglial activation and reduced expression of pro-inflammatory cytokines, which was also associated with neuroprotective effects [321,322]. The inhibition of microglial activation and the observed neuroprotection were reserved upon treatment with CB_1_ selective antagonists, suggesting that the CB_1_ receptor is involved. Although several studies do suggest the anti-inflammatory potential of the CB_1_ receptor, the majority of research is now focused on the CB_2_ receptor in PD. This is due both to the profound upregulation of the CB_2_ receptor on activated microglia, and the results from numerous in vitro studies that demonstrate that activation of the microglial CB_2_ receptor changes the activation state of the microglia, reducing their release of pro-inflammatory cytokines and increasing the release of anti-inflammatory cytokines (reviewed in [323]).

The first study to provide solid evidence that the anti-inflammatory effects of cannabinoid drugs were mediated by the CB_2_ receptor was carried out in an MPTP model in mice. The non-selective cannabinoid agonist WIN55,212-2 reduced MPTP-induced microglial CD11b marker upregulation in the ventral midbrain, and this effect was blocked by the CB_2_ antagonist JTE-907 [324]. In the decade since the publication of this paper, several studies investigating CB_2_ receptors in animal models of PD have reported an anti-inflammatory effect. Garcia and colleagues found that chronic administration of Δ9-THCV attenuated the loss of dopaminergic neurons in both the 6-OHDA and LPS model. This effect was also elicited in the LPS model by the CB_2_ selective agonist HU-308, suggesting that the effect was CB_2_ receptor-mediated [325]. However, HU-308 did not induce a significant neuroprotective effect in 6-OHDA lesioned rats [326]. It is possible that the reason for this is the lower inflammatory response induced by 6-OHDA, which is a direct neurotoxin, compared to LPS, an endotoxin expressed in the outer membrane of gram-negative bacteria, which evokes an immune response. Treatment with the naturally occurring CB_2_ agonist β-caryophyllene has also been demonstrated to reduce rotenone-induced dopaminergic degeneration and microglial activation [327].

An exacerbation of MPTP-induced toxicity has been described in CB_2_ knockout mice [324]. An aggravation of inflammation and neuronal death due to genetic inactivation of the CB_2_ receptors has also been reported in the LPS model, but not in the 6-OHDA model [313,325]. In contrast, mice overexpressing CB_2_ receptors present significantly less motor impairment compared to wild-type mice following intra-caudate 6-OHDA administration, as well as reduced microgliosis and astrocytosis [328].

Alterations in CB_2_ receptor expression have been seen in animal models of PD induced by a variety of stimuli including 6-OHDA, rotenone, LPS, and poly I:C. All these neurotoxins caused a marked upregulation in CB_2_ receptor expression in the rat striatum, peaking two weeks post-lesion [171,175]. Interestingly, a more pronounced upregulation was observed in response to the bacterial and viral inflammagens, LPS and poly I:C respectively, compared to the direct neurotoxins. In the 6-OHDA, rotenone, and LPS models, CB_2_ receptor expression correlated strongly with expression of the microglial marker CD11b. Similar co-expression of CD11b (Mac1) and CB_2_ immunostaining was observed in the ventral midbrain of mice three days after treatment with MPTP, a dopamine-selective neurotoxin [324]. An upregulation of CB_2_ expression on microglia has also been confirmed to be present in post-mortem PD brains [313]. However, it is not only on microglia that CB_2_ expression is altered in PD. Tyrosine hydroxylase (TH)-positive neurons in the substantia nigra have been demonstrated to express CB_2_ receptors, and in PD there is significantly reduced labeling of the receptor, in concordance with the loss of these dopaminergic neurons in the disease, but also seemingly a reduced expression by surviving cells [312].

The majority of the information presented in this review is pre-clinical, and the animal models of PD used to evaluate neuroinflammation are limited to toxin models. Future studies are necessary to extend evaluation of cannabinoids to other PD models that incorporate inflammation such as those with an environmental and a genetic interaction. Additionally, with the growing interest in the dysfunctional communication between the peripheral and central immune systems in disease, studies should discern cannabinoid-mediated effects on the crosstalk and the infiltration of peripheral immune cells. The number of clinical trials assessing cannabinoids and their effect on inflammation are limited, and non-existent in the context of neuroinflammation in neurodegenerative disease. While pre-clinical trials are necessary to answer a number of questions that remain regarding the molecular mechanisms of cannabinoids in relation to microglial phenotypes and neuroinflammation, there is also a need for clinical trials to assess the safety and efficacy of various cannabinoid compounds for PD.

## 5. Summary and Conclusions

Microglia play a crucial role in the uninjured brain, carrying out homeostatic maintenance and constantly monitoring their local microenvironment for indications of danger. Their activation and polarization in response to a harmful stimulus is essential in order to resolve an acute injury, but the chronic overactivation and dysregulation of microglia can lead to a persistent cycle of neuroinflammation and neuronal death in neurodegenerative diseases such as PD. Cannabinoids have the potential to modulate the activity of microglia, shifting them towards a less cytotoxic phenotype. From a clinical perspective, this suggests that administration of exogenous cannabinoids or molecules that increase endogenous cannabinoids could produce anti-inflammatory effects through cannabinoid actions on microglia, and could thus decelerate the progression of PD.

## Figures and Tables

**Table 1:** Cannabinoid-mediated effects on immune cell function of primary microglial cell cultures. ↑, increase. ↓, decrease. LPS, lipopolysaccharide. CB_1/2_ non-selective agonist: CP55, 940, WIN55,212-2, HU-210. CB_1_ antagonist: AM251. CB_2_ selective agonist: JWH133, JWH015. CB_2_ selective antagonist: AM630. CB_2_ inverse agonist: SMM-189, SR144528.

Species	Inflammagen	CB Treatment	Microglial Effects	Reference
CB_2_^−/−^ mice (Deltagen, Jackson Lab)—C57BL/6	IL-4 + IL-13		↓ Arg1 in KO microglia than WT microglia	[272]
	IL-4 + IL-13		↓ Phagocytic activity in KO microglia measured by reduced engulfed fluorescent microspheres	
Rat (P0–P2)—Wistar	IL-4 + IL-13		↑ Arg1 mRNA and protein ↑ Acute (6 hr) in mRNA of CB_2_ and DAGLα ↓ Prolonged (24 hr) mRNA in MAGL, FAAH, and ↑ in CB_1_ and CB_2_, and NAPE-PLD	[272]
	IL-4 + IL-13	AM251 or AM630	↓ Cytokine-induced Arg1 mRNA and protein	
	TGF-β		↑ Acute (6 hr) in mRNA of CB_1_, CB_2_, NAPE-PLD, DAGLβ, and MAGL and ↓ FAAH Prolonged (24 hr) mRNA ↓ in NAPE-PLD, MAGL, and FAAH	
	LPS		Acute (6 hr) ↓ in CB_1_, CB_2_, FAAH, NAPE-PLD, and MAGL with prolonged (24 hr) ↓ in FAAH and MAGL	
Rat (P1–P2)—Sprague Dawley	LPS	AEA	↓ LPS-induced microglia cytokine mRNA of IL-1α and TNF	[287]
	LPS	SR144,528	↑ Microglia cytokine mRNA in dose-dependent manner (IL-1α, IL-1β, IL-6, TNF)	
Rat cortical (neonate)—Sprague Dawley	LPS	CP55,940	↓ LPS-induced production of TNFα	[288]
	LPS	AEA or 2-AG	↓ LPS-induced production of TNFα	
Rat cortical (P2–P3)—Sprague Dawley	LPS	JWH015	↓ LPS-induced TNF protein expression ↓ LPS-stimulated microglia chemotaxis measured by # of cells that migrated toward the chemoattractant ADP	[289]
	LPS	JWH015 + AM630	JWH015 effect on chemotaxis blocked by AM630, demonstrating CB_2_-specific effect on microglia migration	
Mouse forebrain (newborn)—Balb/c	LPS and IFNγ	Anandamide	↑ Further the LPS/IFNγ-induced expression of IL-10	[291]
	LPS and IFNγ	JWH133	↑ Further the LPS/IFNγ-induced expression of IL-10 that is ↓ with SR144528 (CB_2_ inverse agonist) and not by SR141716A (CB_1_ antagonist), suggesting CB_2_-mediated mechanism	
Rat cortical (neonate)	Fibrillar βA_25–35_	HU-210, WIN55,212-2, or JWH133	↓ Fibrillar βA-induced TNF microglial release	[290]
Human	LPS	SMM-189	↓ IFN-γ, IL-6, IL-12p70, and chemokines IL-8, MCP-1, CCL17 (TARC), macrophage derived-chemokine (MDC), and eotaxin-3	[292]

**Table 2:** Cannabinoid-mediated effects on microglia/inflammation and neuroprotection in models of Parkinson’s disease. ↑, increase. ↓, decrease. ICV, intracerebroventricular. 8-OHdG, 8-hydroxy-2- deoxyguanosine. LPS, lipopolysaccharide. MFB, medial forebrain bundle. CB_1/2_ non-selective agonist: WIN55,212-2, HU-210. CB_1_ antagonist: AM251, SR141716A. CB_2_ selective agonist: HU-308, JWH015. CB_2_ selective antagonist: JTE907, AM630. CB_2_ inverse agonist: SMM-189, SR144528.

Species	Inflammagen	Cannabinoid Treatment	Treatment Timeline	Microglia/Inflammation Effects	Neuroprotective Effect	Reference
C57BL/6 mice	MPTP (4 × 20 mg/kg every 2 hrs)	HU-210 or WIN55,212-2	2 d before MPTP and again 12 hrs after MPTP and continue for 3 d (microglia analysis) and 7 d (neuron analysis)	↓ MPTP-induced nigral Mac1 (CD11b) activation and production of O2−(ethidium accumulation) ↓ MPTP-induced 8-OHdG suggesting reduced protein oxidative damage ↓ MPTP-induced nigral TNF, IL-1β, and *iNOS* gene expression and TNF and IL-1β protein expression	↑ MPTP-induced TH+ stereological nigral cell count ↑ MPTP-induced rotarod latency to fall and striatal dopamine content	[322]
		+AM251 (microglia/ neuron) or SR141716A (neuron)	30 min prior to non-selective agonists	↑ Agonist-induced nigral Mac1 (CD11b) activation and production of O_2_^−^ (ethidium accumulation) ↑ Agonist-induced 8-OHdG suggesting oxidative damage ↑ Agonist-induced nigral TNF, IL-1β, and *iNOS* gene expression and TNF and IL-1β protein expression	↓ Agonist-induced TH+ stereological nigral cell count (no difference with MPTP alone group) when treated with CB_1_ antagonists ↓ Agonist-induced rotarod latency to fall and striatal dopamine content (no difference with MPTP alone group) when treated with AM251	
CB_2_^−/−^ (C57BL/6 background)	Intrastriatal LPS	-	-	↑ CD68-immunoreactivity in the nigra of KO mice compared to WT	Not evaluated	[313]
C57BL/6 mice	Intrastriatal LPS	HU-308	Daily injections for 2 weeks starting 16 hr after LPS	↓ LPS-induced nigral CD68-immunoreactivity ↓ LPS-induced striatal *iNOS* gene expression	↑ LPS-induced nigral TH-immunoreactivity	
C57BL/6 P1 glia and cerebellar neural cultures	6-OHDA	HU-210 directly to cultures	Neurons +/− conditioned media from glia culture 24 hrs after HU-210	Not directly evaluated but neuroprotective effects from glia conditioned media suggest CB_1_ and CB_2_-mediated glial effects	↑ cerebellar granule cell survival with direct HU-210 to neurons and greater protection when neurons treated with glia conditioned media treated with HU-210	[320]
Sprague Dawley rats	Intranigral LPS	HU-210 or WIN55,212-2	ICV injections 1 hr prior to LPS	↓ LPS-induced nigral CD11b activation and production of O_2_^−^ (ethidium accumulation) ↓ TNF and IL-1β after WIN55,212-2 and ↓ IL-1β after HU-210 24 hrs after LPS as measured by ELISA ↓ p67phox and p47phox subunits in cytosol and membrane nigral fractions 12 hrs after LPS by western, suggesting reduced translocation of NADPH oxidase which was specific to CD11b^+^ cells	↑ TH+ stereological nigral cell count	[321]
C57BL/6 mice	MPTP (4 × 20 mg/kg every 2 hrs)	JWH015 (microglia) or WIN55,212-2 (microglia/ neuron)	Daily for 3d (microglia analysis) or 5 d (neuron analysis) starting 1 d after MPTP	↓ MPTP-induced nigral Mac1 (CD11b) protein with WIN55,212-2	↑ MPTP-induced nigral TH^+^ stereological neuron counts (dose-dependent) ↑ MPTP-induced midbrain dopamine levels after WIN55,212-2 treatment	[324]
		WIN55,212-2 +JTE907	20 min before WIN55,212-2	↑ Agonist-induced Mac1 (CD11b) when administered alone or in conjunction with WIN55,212-2 agonist	Not evaluated	
Sprague Dawley rats	ICV 6-OHDA	Δ9-THCV or enriched CBD	Daily for 14 d starting 16 hrs after 6-OHDA	↓ 6-OHDA-induced nigral OX-42-immunoreactivity with either Δ9-THCV or enriched CBD	↑ 6-OHDA-induced nigral TH-immunoreactivity when treated with CBD but not Δ9-THCV	[325]
C57BL/6 mice	Intrastriatal LPS	Δ9-THCV or HU-308	Daily for 14 d starting 16 hrs after LPS	Not evaluated	↑ LPS-induced nigral TH-immunoreactivity when treated with HU-308 or Δ9-THCV	
Sprague Dawley rats	Unilateral MFB injection of 6-OHDA	CBD	Daily for 2 weeks, starting 16 hr after 6-OHDA	↑ 6-OHDA induced striatal *Cu*,*Zn-SOD* gene expression suggesting a protection from endogenous oxidative stress	Not evaluated	[326]
		HU-308	Daily for 2 weeks, starting 16 hr after 6-OHDA	Not evaluated	Did not alter striatal TH activity by HPLC or nigral TH mRNA levels compared to 6-OHDA+vehicle	
Wistar rats	Rotenone i.p. once daily for 4 weeks	βcaryophyllene (BCP) ± AM630	Daily for 4 weeks and 30 min prior to rotenone	↓ Rotenone-induced striatal Iba1+ activated microglia with BCP and blocked by AM630 ↓ Rotenone-induced striatal GFAP activated astrocytes with BCP and blocked by AM630 ↓ Rotenone-induced midbrain pro-inflammatory cytokines IL-1β, TNF, and IL-6 with BCP and blocked by AM630 ↓ Rotenone-induced striatal NFκB p65, COX-2, iNOS with BCP and blocked by AM630	↑ Rotenone-induced striatal and nigral TH-immunoreactivity with BCP and blocked by AM630	[327]
CB2xP mice (overexpression of mouse CB_2_)	Unilateral striatal 6-OHDA	-	7 weeks	↓ 6-OHDA-induced striatal GFAP expression vs. WT Overexpression of CB_2_ altered striatal Iba1 immunoreactivity, but not striatal levels of iNOS and COX2 vs. matched WT ↓ Striatal malonyldialdehyde (lipid peroxidation product) vs. WT at basal and 6-OHDA conditions ↓ 6-OHDA induced striatal ratio of oxidized GSSG:glutathione (oxidative stress marker) vs. WT	↓ 6-OHDA-induced apomorphine rotations vs. WT ↑ 6-OHDA-induced time in open arms of elevated plus maze suggesting CB_2_ role in anxiety-like behavior vs. WT ↑ 6-OHDA-induced memory impairment in step-down inhibitory avoidance task vs. WT ↑ 6-OHDA-induced striatal and nigral TH immunostaining vs. WT	[328]
